# Supplementary figures and images for: Four layer multi-omics reveals molecular responses to aneuploidy in Leishmania
Source: PLoS Pathog. 2022 Sep 23;18(9):e1010848. doi: 10.1371/journal.ppat.1010848 (PMC9534393; doi:10.1371/journal.ppat.1010848)

Supplementary Figures


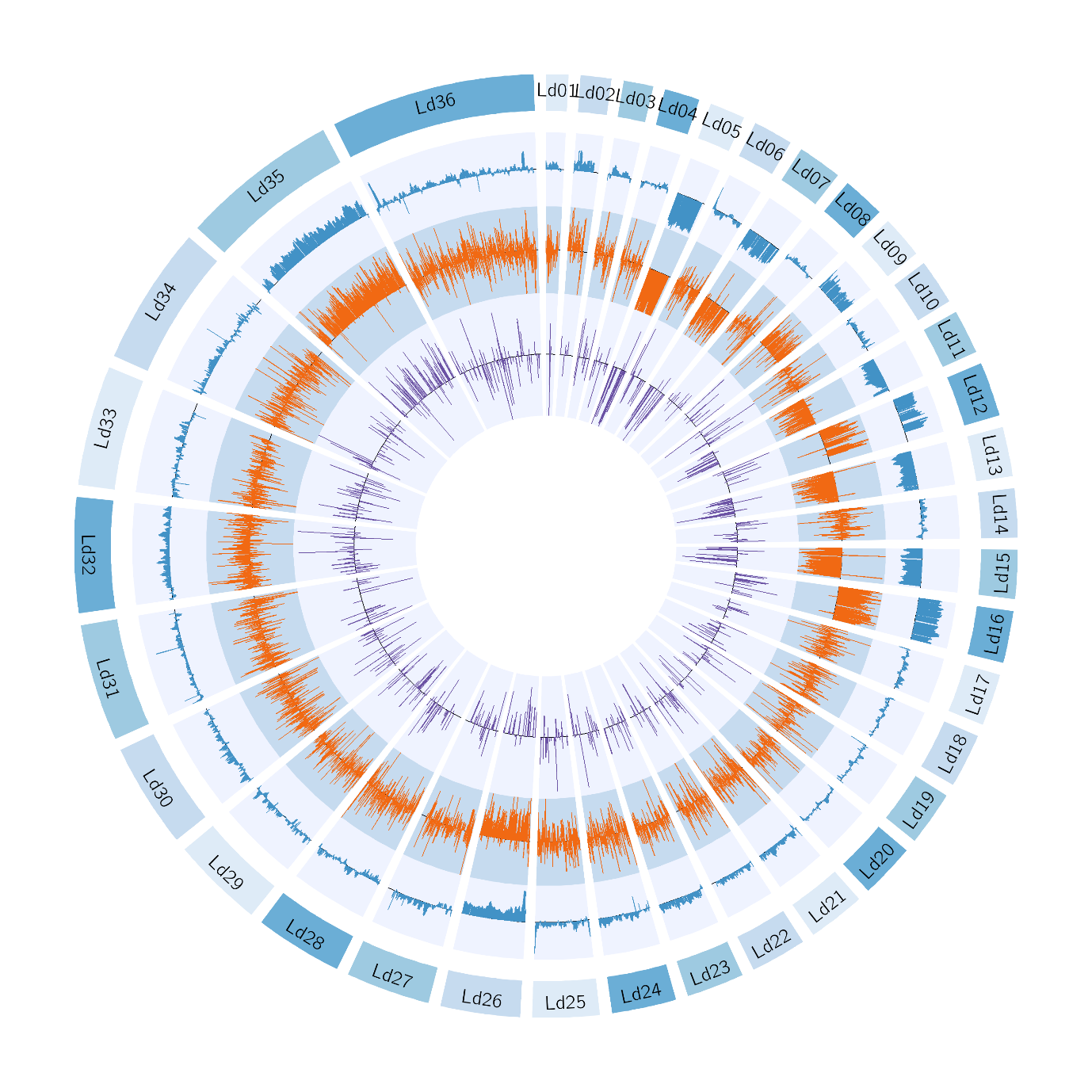


Fig S1A: Circos BPK173 vs BPK288


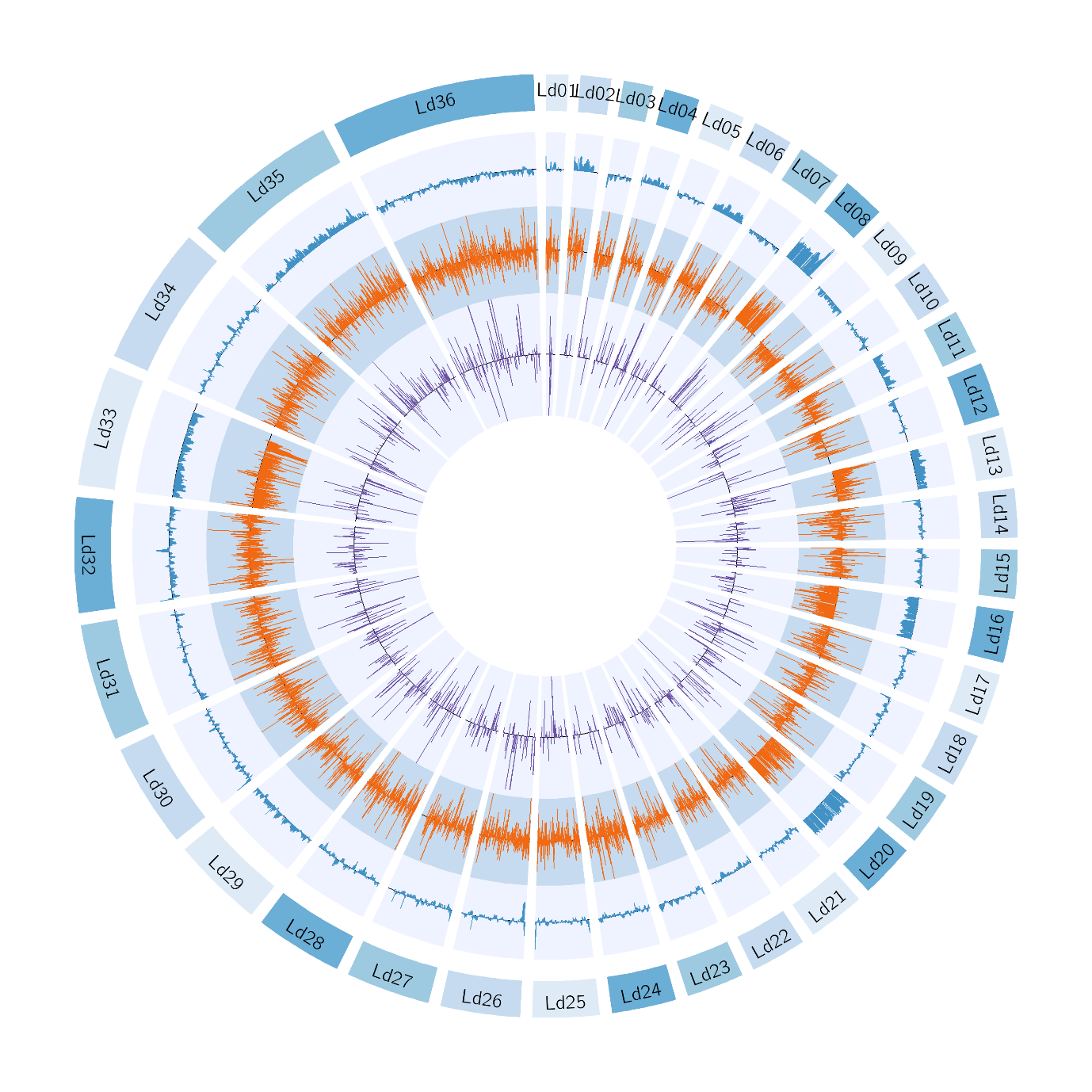


Fig S1B: Circos BHU575 vs BPK282

Supplement: S1 Fig — Comparative Circos plot between BPK173 and BPK288 (S1A) and between BHU575 and BPK282 (S1B), showing their relative (expressed in fold change) gene dosage (blue), transcript abundance (orange), and protein abundance (purple) across the 36 L. donovani chromosomes. (DOCX) [file ppat.1010848.s006.docx]
