# Supplementary material for: Four layer multi-omics reveals molecular responses to aneuploidy in Leishmania
Source: PLoS Pathog. 2022 Sep 23;18(9):e1010848. doi: 10.1371/journal.ppat.1010848 (PMC9534393; doi:10.1371/journal.ppat.1010848)
Supplement: Attachment — Submitted filename: 121334_1_rebuttal_2384072_rdf9q3-1.docx [file ppat.1010848.s007.docx]

**Manuscript number:** RC-2021-01110R

**Corresponding author(s):** dr. Bart Cuypers, prof. dr. Jean-Claude Dujardin, dr. Malgorzata Domagalska, prof. dr. Kris Laukens.

## 1. General Statements [optional]

NA

## 2. Point-by-point description of the revisions

Comments of editor and reviewers are numbered. Our response is preceded by R and is in italics. When relevant we pasted the modified text of the ms: this is marked in bold and we mention the page and lines where this text can be found (track change version)

**Reviewer #1** (Evidence, reproducibility and clarity (Required)):

Review of the manuscript by Cuypers et al "Four layer multi-omics reveals molecular responses to aneuploidy in Leishmania"

The parasitic organism Leishmania donovani is known to exist with aneuploid chromosomal content, often as a mosaic of various aneuploid karyotypes. The genomic instability and subsequent aneuploidy may explain its ability to select advantageous karyotypes in changing environments, however, the consequences of aneuploidy have not been carefully evaluated yet. The gene expression in Leishmani occurs in polycistronic units, and thus regulation of protein abundance is entirely posttranscriptional. The study presents integrated genome, transcriptome, proteome and metabolome analysis of six L. donovani strains and suggests that the metabolic differences between strains may be explained by the underlying aneuploidy. They show that the chromosome copy number results in closely correlated changes in transcripts' abundances and similar, but less correlated changes in protein abundance. This corresponds well with all previously published data on the effects of aneuploidy on gene expression. Analysis of metabolome revealed a correlation between karyotype differences and metabolite levels, although no causal links could be inferred.

**1.1** The strategy they used for normalization remains unclear. In the Fig. A, they present normalize transcript and protein expression, but it remains unclear how exactly they normalized it. There is no normalization step explained in material and methods. This has to be clarified. If the manuscript focuses on the changes in gene expression, then the normalization steps have to be carefully described.

*R. We thank the reviewer for this comment and agree that we should have explained the normalization procedure better. We have now added a detailed description of the transcript and protein expression normalization procedure in the methods section. Additionally, we have made the code publicly available on GitHub.*

***Methods L206-L215: “Average transcript and protein abundance levels per chromosome per strain (Fig 1 C): First, the median was taken of the Log2 normalized transcript counts (see respective section) and Log2 LFQ protein values, per chromosome per strain. This yielded an expression value for each chromosome in each strain, for both transcript and protein expression. Then, for each chromosome, the median of the expression values of all strains that were disomic (determined based on DNA sequencing data) for that chromosome was selected as the ‘disomic reference value’ for that chromosome (i.e., the expression value that corresponds to the median expression of a transcript/protein on a disomic chromosome). The remaining per-strain per-chromosome expression were then divided by this disomic reference value, multiplied by 2, and plotted in Figure 1C.*** ***The code for these normalizations and analyses is available at (***[***https://github.com/CuypersBart/Leishmania_Multiomics***](https://github.com/CuypersBart/Leishmania_Multiomics)***).***

**1.2** The same applies to the dosage compensation of protein abundance - it is difficult to judge unless the exact approach of normalization is explained.

*R. We have now explained the normalization procedure in the methods and uploaded the normalization code to GitHub.*

***Methods L221-L230: “Dosage compensation analysis (Figure 2): This analysis was carried out on the only three chromosomes (Ld05, Ld08, and Ld33) that were at least once disomic (somy of 1.7-2.3), trisomic (somy of 2.7-3.3), and tetrasomic (somy of 3.5 or more) in our six study strains and had more than 10 detected proteins. The abundance values of transcripts (corrected for library size, see above) and proteins (LFQ) were divided by their abundance in the strain that was the most disomic for their encoding chromosome, according to the genome sequencing data. These expression values were then multiplied by 2 so that the normalized abundance at disomy = 2. Strains were always normalized by another strain than themselves to avoid underestimation of the standard deviation. Therefore, we do not have disomic distributions for Ld08 since Ld08 was exclusively disomic in BPK282, and thus already was used as reference strain.”***

**1.3** Some statements are generalized and nor properly explained, for example the statement on line 376 : Strikingly, 12.2% (22/180) of all trans-proteins concerned subunits of macromolecular complexes. This might be potentially interesting, but we do not know how many of all proteins are subunits of macromolecular complexes in Leishmania. Maybe it is also around 12 % and then this statement would not be interesting and striking at all. However, this information is not disclosed. There are more similar examples.

*R. We agree with the reviewer. We should have statistically compared the compensated proteins to the relevant reference set to verify the significance of our observation, i.e. the enrichment of protein complex subunits in this set of compensated proteins. However, in this case, the whole genome is not a good reference set, as there might be a bias in which proteins get measured by the LC-MS proteomics or were excluded for the analysis because of their high variance. Therefore, we checked for significance comparing with the non-compensated proteins.*

***Results L344-L352: “We noted that within the top 5 most compensated proteins in this high-accuracy set, 2 coded for subunits of protein complexes: e.g., the cleavage and Polyadenylation Specificity Factor (CPSF) subunit A and the dynein light chain had respectively 1.12 and 0.96 Log2FC units lower protein than transcript abundance. Accordingly, we hypothesized that protein complexes might be more likely to be dosage compensated (>= 0.30 Log2FC units). To test this systematically, all 85 proteins were screened for their involvement in heteromeric protein complexes (using annotation keywords ‘subunit’ and ‘chain’, Suppl. Table S1.E). Strikingly, complex subunits accounted for 15.6 % (5 out of 32) of the compensated, proteins which was significantly more than the 1.8% (1 out of 53) we observed for the uncompensated proteins (Fischer exact p = 0.0265).”***

*Similarly, statistical backing was missing in our functional description of the trans-proteins, which has now been added.*

***Results L409-L414: “We then compared if these keywords were significantly enriched versus all detected proteins with a Fisher Exact test. 7 of these were indeed significantly enriched of which 3 related to protein metabolism: peptidase activity (Adj. p = 9.4 x 10-5), heat-shock protein (Adj. p = 2.3 x 10-2) and chaperon(in) (Adj. p = 2.1 x 10-2), 2 related to metabolism: glutamate (Adj. p = 1.9 x 10-2) and long-chain-fatty-acid-CoA ligase (Adj. p = 5.4 x 10-3), and two others: mitochondrial (Adj. p = 3.0 x 10-2) and hypothetical protein (FDR adjusted p = 3.4 x 10-7).”***

**1.4.** I understand that it is difficult to analyze the pathways when the genes are not annotated. However, in this form it is not very useful that they performed the transcriptomics and proteomics, but did only limited analysis. The authors should use the functional annotation workstation Blast2GO, which can be really helpful (<https://www.blast2go.com/>).

*R. We believe there is a misunderstanding here and take full responsibility for this. We wrote in our manuscript “Overrepresentation analysis showed that no GO terms were significantly enriched in the pooled list of trans-transcripts or trans-proteins from all comparisons (FDR adjusted p threshold 0.05),* ***but it must be noted here that GO- and other annotations in Leishmania are very poor*** *due to its distinct evolutionary position compared to better studied model organisms.” With ‘very poor’ we did not mean that annotation efforts have been poor as the reviewer interpreted. In fact, the reference genome we used has been extensively and functionally annotated (including with GO terms) to the current state-of-the art, as is explained in Dumetz et al., mBio 2017 (PMID: 28536289). Instead, we meant that the distinct Eukaryotes like Leishmania have little homology with (much) better-studied model organisms. As such, the annotations pipelines, which are homology-based can only transfer annotations to a limited extend (e.g. Leishmania has many hypothetical proteins) or only to a superficial level (e.g. an annotated DNA-binding domain, but the function of the protein being further unknown).*

*We now clarified this in the manuscript:*

***Results L403-L406:*** ***However, it must be noted here that GO- and other annotations in Leishmania are often relatively unspecific due to the parasite’s distinct evolutionary position, and low gene homology with better studied model organisms. For example, only 48.7% of the L. donovani genes could be annotated with at least one GO term, and even these are often of the highest hierarchies (low specificity)^11^.***

*Additionally, we now also use an alternative enrichment analysis strategy, which did yield significant results and is detailed below under comment 6 of this reviewer.*

**1.5.** They claim that Leishmania is unique in the sense that genome-wide response to aneuploidy does not affect transcriptome in trans, but the effects can be seen on proteome. This is indeed unique, however, given that Leishmania generally largely regulates genes expression posttranscriptionally, due to its unusual polycistronic organization of genes, it is not surprising - Leishmania just simply cannot do it differently. From this point of view it is really impressive that there are some changes on proteome nevertheless, suggesting that aneuploidy triggers some response no matter what is the mechanism of regulation of expression.

*R. We thank the reviewer for this comment and agree. We now frame this context much more elaborately in the discussion and summarize it in the conclusion*.

***Discussion L561-L570:* “*Strikingly, we did not observe this coordinated transcriptomic response in Leishmania, with relatively low proportions of differential trans-transcripts between aneuploid strains and no significant enrichment of specific functional classes amongst these. On the one hand, this matches the constitutive gene abundance model of the parasite, where the transcription of individual protein-coding genes can not be controlled by transcription factors. On the other hand, Leishmania does feature substantial post-transcriptional regulation of transcript levels (e.g. between life stages), so in theory, extensive regulation in response to aneuploidy could have occurred at this level, but we did not observe this. Instead, we did find a surprisingly high proportion of trans-proteins. It appears that trans-protein abundances are directly modulated at the translation or protein-stability level, as the majority did not have underlying transcript-level changes.”***

**1.6.** The authors mention (line 379): Further, we found many trans-proteins to be involved in protein metabolism, transport and metabolic functions. This is potentially very interesting, as protein metabolism, transport and metabolic functions were altered in response to aneuploidy in other organisms. Yet, they make no effort to elaborate even a little bit. What protein metabolism proteins? Which metabolic functions? This would be extremely useful.

*R.* *We agree that providing a more detailed analysis of the trans-proteins is important. Cfr comment 1.3 of this reviewer we completely revised this section and aside from more detail, now also provide statistical support for the enriched protein classes amongst trans-proteins.*

***Results L410-L418: “7 of these were indeed significantly enriched of which 3 related to protein metabolism: peptidase activity (Adj. p = 9.4 x 10-5), heat-shock protein (Adj. p = 2.3 x 10-2) and chaperon(in) (Adj. p = 2.1 x 10-2), 2 related to metabolism: glutamate (Adj. p = 1.9 x 10-2) and long-chain-fatty-acid-CoA ligase (Adj. p = 5.4 x 10-3), and two others: mitochondrial (Adj. p = 3.0 x 10-2) and hypothetical protein (FDR adjusted p = 3.4 x 10-7). At RNA-level none of the keywords were significant. This demonstrates that differential trans-proteins associated with aneuploidy are not a random occurrences, but related to specific functional classes and processes. Particularly relevant in this context are the enrichment of heat-shock proteins and chaperones, as aneuploidy is known to burden protein folding pathways^50^.”***

*The full list of trans-proteins is also provided in Supplementary Table 1H.*

**1.7.** The authors state (line 457): We confirmed that the transcript abundance of aneuploid chromosomes is globally affected. This is unclear. They explained in the Results that transcripts are affect only for the aneuploid chromosome, and trans changes are very small, much smaller than for proteins. It is unclear how they came to this statement in Discussion.

*R. We believe there is a misunderstanding here. With the sentence “We confirmed that the transcript abundance of* ***aneuploid*** *chromosomes is globally affected”, we mean exactly what the reviewer says: “They explained in the Results that transcripts are affect only for the aneuploid chromosome”. We do not make any statement about trans transcriptional changes here.

To avoid any future confusion for readers, we have modified the sentence to:*

**Discussion L494: *“We confirmed that transcript abundance is affected across the entirety of aneuploid chromosomes”***

**1.Final** Reviewer #1 (Significance (Required)):

Altogether, this is a potentially interesting study, as Leishmania and other parasites probably use aneuploidy as an evolutionary strategy for fast adaptation to their changing environment. However, as presented here the study has a limited novelty and no unique insight. Most of the conclusion that can be made from the partially analyzed data have been known. The only unique observation related to the largely posttranscriptional global changes is interesting, although it is apparent that this is the way how gene expression regulation functions in Leishmania. The study is also only half finished, as only the basic analyses were performed and only little was tried beyond the obvious.

*R. We thank the reviewer for the assessment of our manuscript and valuable input.

Firstly, we are a sorry that it was not clear from the first version what the novelty of the manuscript was. We believe that we have clarified this better in the new version what the novelty is for Leishmania, and for other Eukaryotes.*

*Novelty for Leishmania:*

***Introduction L74-L78: “While aneuploidy in Leishmania is known to directly affect the transcription of encoded transcripts, its molecular impact further downstream remains poorly understood^11,16^. Here we present the first study that generated and integrated genome, transcriptome, proteome, and metabolome data of highly aneuploid L. donovani strains, in vitro.”***

***Introduction L87-88: “Through this integrated 4-‘omic approach, we provide a systems-wide view of the molecular changes associated with aneuploidy in Leishmania.”***

*As such, we are the first to describe the strong link between aneuploidy-driven transcript abundance and protein abundance, and between both layers and the parasite’s metabolome****.*** *We are also to first to describe dosage-compensation and trans-effects in Leishmania.*

*Novelty for other Eukaryotes:*

***Conclusion L593-L602: “While most aneuploid Eukaryotes display extensive regulation of trans-transcripts to mediate the effects of aneuploidy, we did not observe this to the same extend in Leishmania. This suggests that the post-transcriptional mRNA regulation system of the parasite does not mediate the impact of aneuploidy. Instead, Leishmania features a surprisingly high number of trans-proteins, which are likely post-transcriptionally modulated. It thus seems that aneuploidy is accompanied by trans-proteomic changes in Eukaryotes, regardless of the system of regulation. Post-transcriptional modulation of trans-proteins may also be present in other Eukaryotes, but could be overshadowed by the transcription factor-induced gene regulation that is absent in Leishmania. For this reason, we believe that Leishmania is an attractive model to study processes of post-transcriptional modulation of protein levels in the context of aneuploidy and beyond.”***

*Secondly, we agree with the reviewer that some of our analysis could be considered half-finished as some observations lacked statistical support or could have been more in depth. We have now addressed in the new version of the manuscript. These changes have been detailed above in reviewer comments 1.3 (statistical support for the enrichment of protein complex subunits amongst compensated proteins) and 1.6 (deeper functional analysis and statistical support for trans-proteins).*

**Reviewer #2** (Evidence, reproducibility and clarity (Required)):

This study examines the expression of transcripts, proteins, and metabolites in aneuploids of Leishmania. This case is particularly interesting because Leishmania has an unusual gene regulatory system in which genes are constitutive and polycistronic and so it is of interest whether the usual constraints of aneuploidy on viability and gene expression are operating. Indeed, Leishmania has a high degree of aneuploidy in populations, which might be consistent with this idea. It should be noted, however, that with a chromosome number of 35, each aneuploid would consist of a small fraction of the genome.

**2.1** The authors contend that the major effects of modulation of expression on the transcript and protein levels come from the cis effects of varying individual chromosomes and that modulations across the genome are minimal compared to aneuploidy in other organisms.

*R. This is indeed an important point of difference with other Eukaroytes, which we believe is very relevant for the broader readership.*

**2.2** The authors perform a normalization of their transcriptome analysis to the geometric mean of all counts, which could have an effect on the determination of trans effects, particularly negative correlations with the varied chromosome, a common effect of aneuploidy in other species. However, this might not be a major problem considering that the chromosomes analyzed were on the smaller size among the 35 chromosomes in this species.

*R. We thank the reviewer for this comment. We normalized with the geometric mean as it is less sensitive to outliers (very low- or highly expressed genes) than the arithmetic mean.

Regarding the validity of normalizing with the geometric mean count: The most aneuploid strain in our analysis was BPK288 with 12 aneuploid chromosomes. Like the reviewer mentions, this concerned mostly the smaller chromosome (e.g. in the case of BPK288 Ld02, Ld05, Ld07, Ld08, Ld09, Ld11, Ld13, Ld15, Ld20, Ld23, Ld26, Ld33 out of 36 chromosomes that are ranked by increasing size as can be seen on the Circos in Figure 1B). Thus, the vast majority of transcript have a stable expression between conditions which is the requirement for a valid differential expression analysis.*

**2.3** Single celled organisms are subjected to selection pressures that could select changes in absolute dosage effects to increase flux through certain pathways (Conant and Wolfe, 2007; Mol Syst Biol 3:129) and this is certainly a possible scenario in Leishmania. While the authors tend to focus on this direction, there are some clues in the manuscript that the situation is potentially more complex and that some parallels with other organisms might be operating. First, the authors note that the aneuploid strains that they analyzed were aneuploid for chromosomes in the smaller range for this species, which already has a heavily subdivided genome among its chromosomes. Thus, there might be a selection against aneuploidy for larger chromosomes similarly to other organisms. Chromosome 21 in humans has the least number of genes and is the least severe autosomal aneuploid. Then, even for these small chromosomes, affected transcripts off of the varied chromosome were found to be differentially expressed with a range from 13 % to 59% (Table 1). The authors average these to 28% but this is a misleading calculation because each aneuploid would have a different gene content and would be expected to be potentially different, which in fact they appear to be quite dramatically different.

*R. We thank the reviewer for this helpful comments. As for the first point, we believe the parallel the reviewer remarks regarding the more frequent aneuploidy of smaller chromosomes in other Eukaryotes is very interesting. Therefore, we added the following section to the discussion:*

***Discussion L520-L525: “Possibly, this stochiometric imbalance caused by aneuploidy is somehow detrimental to the parasite. One argument in this direction is that we and previous studies found smaller Leishmania chromosomes to be more frequently aneuploid, which matches observation in yeast and humans (chromosome 21 has the smallest number of genes in humans and the least severe autosomal aneuploid) ^10,23,58^. Additionally, it has been shown in yeast that genes that are deleterious when overexpressed are enriched for protein complex subunits^59^.”***	*For the second point (misleading calculation), we believe there is a misunderstanding because of our unclear table legend, as we do take chromosome size into account (indirectly). The percentages are normalized to the total number of transcript/protein changes in that comparison, which are smaller for small aneuploid chromosomes, and larger for large aneuploid chromosomes. We have now clarified the table legend.*

**Results L419-422: “*Table 1: Total numbers (N) of differential (FDR adjusted p < 0.05) transcripts and proteins between all possible pairs of our 6 L.donovani strains. Each time, it is indicated what proportion of these transcripts or proteins originate from chromosomes that have somy changes (because of aneuploidy) in the strain pair (Cis) or chromosomes that have identical somies between the two strains (Trans).”***

**2.4** For proteins, it was noted that members of complexes tended to show "dosage compensation" from the varied chromosome. As the authors point out, subunits of complexes not incorporated into the complex will be degraded, as has long been indicated. Given that steady state levels were analyzed, the result found was to be expected. How differential synthesis of different subunits of multicomponent interactions would impact cellular functions is unknown and not easily addressed in any system.

*R. The reviewer is correct that dosage compensation of protein complexes subunits has previously been indicated, which we also remark in our manuscript and cite 6 relevant articles to support this (Discussion L513-L514). However, we would like to point out it has never been indicated in the Leishmania species, the Trypanosomatid family or even in the Euglenozoa phylum. Not all findings from human, yeast or plant species can be extrapolated to other Eukaryotic phyla. Gene expression and regulation often work very different in Euglenozoa, so even if expected, it is important to confirm it and report on the precise way it happens. We strongly believe it is important to report on both the similarities and differences between well-studied Eukaryotes and distinct, understudied Eukaryotic Phyla.*

**2.5** There is an absence of a discussion of a related situation in yeast. Yeast has polymorphism for aneuploidy as well but depending on the genotype, there are fitness consequences. Despite the polymorphism for aneuploidy, there are indications that the stoichiometry of multicomponent interactions or complexes plays a role in yeast. Overexpression studies indicate that those genes with the most reduced fitness tend to be involved in complexes (Robinson et al 2021, eLife 10:e70564). Heterozygotes of vital genes have increasingly reduced fitness with increasing involvement in complexes (Papp et al 2003, Nature 424: 194-197). Duplicate genes from whole genome duplication versus small-scale duplication have different classes of genes that are retained (Hakes et al 2007, Genome Biology 8: R209), which is a typical signature of the impact of members of complexes on selection and with parallels to aneuploidy. Despite the tolerance of aneuploidy in yeast, modulations across the genome occur (Hou et al 2018, PNAS 115: E11321-11330). Thus, the question arises as to whether there are more parallels to aneuploidy in other organisms, particularly unicellular eukaryotes, than suggested in the manuscript.

*R. We would like to point out that we already explicitly mention in our discussion that dosage compensation of protein complex subunits has been observed before in yeast and human, and cite references accordingly (Discussion L513-L514). However, we were not aware that overexpression of genes involved in complexes have a demonstrated fitness cost in yeast. We thank the reviewer for remarking this and have added this to the discussion.*

***Discussion L524-L525: “Additionally, it has been shown in yeast that genes that are deleterious when overexpressed are enriched for protein complex subunits^59^”***

**2.6** The citations to other organisms (mentioned humans, yeast, maize, and Arabidopsis) that have high abundances of trans-transcripts is an interesting set that are not really ones in which the most comprehensive analyses were performed. In terms of protein modulations, the first scan of a large portion of a genome (maize) was Birchler and Newton, 1981, Genetics 99: 247-266. Transcript levels were examined in maize in Guo and Birchler, 1994, Science 266: 1999-2002. Global studies on transcripts in maize are Yang et al 2021, Plant Cell 33: 901-916 and Shi et al 2021, Plant Cell 33: 917-939. Not mentioned in the manuscript is Drosophila where aneuploidy has been examined with trans effects (Devlin et al 1988, Genetics 118; 87-101; Birchler et al 1990, Genetics 124: 677-686; Sun et al 2013, PNAS 110: 7383-7388 & PNAS 110: 16514-16519). A comprehensive study of Arabidopsis is Hou et al 2018, PNAS 115: E11321-11330 with re-analysis of yeast data. Nice studies of human aneuploidy are Raznahan et al 2018 PNAS 115: 7398-7403 and Zhang et la 2020, PNAS 117:4864-4873.

*R. We thank the reviewer for providing these additional references. We have now added Drosophila and several of the suggested references to the manuscript (Sun et al. PNAS 110: 16514-16519, Hou et al. PNAS 115: E11321-11330, Raznahan et al 2018 PNAS 115: 7398-7403 and Zhang et la 2020, PNAS 117:4864-4873).*

**2.7** The unique system of gene expression in Leishmania provides an interesting model to examine the impact of aneuploidy. The authors tend to suggest that there distinctions with other eukaryotes with regard to the impact of aneuploidy and given the gene expression mode, there probably are. However, given the small size of the aneuploids studied and the points raised above, the distinctions might not be as strong as suggested. The situation might be somewhat analogous to yeast. Unicellular organisms would be subjected to selection for increased amounts of certain proteins under some environmental conditions and aneuploidy is one means to produce such an increase. That is not a viable option for multicellular organisms. However, given that aneuploidy for the smaller chromosomes, even among small chromosomes in general, was the most prevalent, there is probably a detrimental effect of aneuploidy in Leishmania also.

*R. We thank the reviewer for this comment. We agree with the argument about the potential detrimentally of aneuploidy and now also include this in the discussion (see also previous comment 2.3 from this reviewer where the relevant sections are cited).*

**2.Final** Reviewer #2 (Significance (Required)):

The system is interesting and addresses an unusual circumstance. Continued study and more in depth bioinformatic and network studies might be quite interesting.

*R. We thank the reviewer for acknowledging the value of our study. We have introduced new bioinformatic analysis and results that were recommended by reviewer 1 (see comment 1.3 and 1.6).*

**2. **Referee Cross-commenting****

I agree with Reviewer #1 that the study is not as well documented as needed and is potentially flawed to some degree. The authors appear to be unaware of a long history of studies on aneuploidy and it is not clear if they are inclined to learn from that history. I don't think the manuscript should be published in its present form and it is not clear the authors have the disposition to adjust the manuscript accordingly. It is a fascinating system in which to ask the posed questions, so a thoughtful and well designed study would certainly be interesting.

*R. Reviewer 1 remarked that some analysis were not enough in depth, some lacked statistical support and also the novelty was not clear. As we explain under* ***1.Final****, these issues have now been resolved.

We also agree that we should have documented better the methods section, which we have resolved (see reviewer comment 1.1 and 1.2) and cite and discuss better the relevant literature, differentiating better what was known, and what is new (see reviewer comments 1.5, 2.3, 2.4, 2.5, 2.6, 2.7).*

*We believe our study was well designed for the following reasons:*

- *We used 6 different aneuploid strains with up to 12 aneuploid chromosomes*
- *This allowed us to study a wide diversity of karyotypes and aneuploid chromosomes*
- *Per strain in LOG phase, we had 4 replicates for transcriptome, 4 for proteome and 4 for metabolome that were taken from independent culture growth flasks (thus 6 * 4 = 24 replicates per ‘omic layer).*
- *For 3 strains we also studied the situation in STAT phase, for which we added an additional 4 replicates in STAT per strain per ‘omic layer.*

*Finally, we agree with the reviewer that Leishmania is a fascinating system for aneuploidy studies, which was our primary motivation for this study. Indeed, the parasite completely lacks the classical transcriptional regulation (pol2 promotors, transcription factors for every gene) present in all other aneuploidy model systems such as yeast, maize, mouse and human.*

**Reviewer #3 (Evidence, reproducibility and clarity (Required)):**

Leishmania donovani are protozoan parasites that can tolerate whole-chromosome aneuploidy remarkably well. Leishmania isolates naturally display mosaic aneuploidy of nearly all chromosomes without overt lethality. Additionally, these parasites exhibit minimal transcriptional regulation due to their unique operon-like chromosomal organization. Thus, most gene expression regulation may take place at the post-transcriptional level. Here, authors conduct a comprehensive analysis of the effects of Leishmania aneuploidy on their transcriptome, proteome, and metabolome. They find that there is protein-level regulation that curbs the effects of aneuploidy, especially for protein complexes and extracellular proteins. They also find significant protein expression changes on non-aneuploid chromosomes and metabolic dysregulation due to aneuploidy.

**3.1** Overall, I believe that the results are clearly-presented and interpreted appropriately. My suggestions for this manuscript are mostly textual:

*R. We thank the reviewer for acknowledging our work.*

**3.2** Regarding secreted proteins - I understand that the P value was less than .05, but as you were only able to analyze two of them, it seems a stretch to conclude that secreted proteins as a class exhibit more dosage compensation than other proteins (pg16).

*R. We agree with this comment of the reviewer. We still believe this observation is interesting to report, but express more caution now in the manuscript. As such, we have added the following sentence to these results:*

***Results L367****:* ***“However, this result needs to be interpreted with caution as the sample size was only 2 proteins.”***	*Additionally, we removed ‘secreted proteins’ from the following statement from the abstract to further avoid generalization.*

***Abstract L32: “However, protein complex subunits, ~~secreted proteins,~~ and non-cytoplasmic proteins, responded less or even not at all to aneuploidy-induced dosage changes”***

**3.3** "Dosage compensation at the transcript level is absent" is a strong statement, and I do not think there is enough evidence to show there is NO transcriptional dosage compensation. Previously, you showed that transcription levels mirror chromosome copy number at 0.94x the somy. So there may be 6% dosage compensation at the transcriptional level. Perhaps "dosage compensation at the transcript level is minimal or absent" would be better (pg13).

*R. We fully agree and thank the reviewer for this comment. We adapted the sentence exactly as the reviewer proposed.*

***Results L310-311: “This suggests that a somy change affects most transcripts equally, and dosage compensation at the transcript level is minimal or absent.”***

**3.4** When looking at the factors influencing protein dosage compensation, the authors should correct for multiple hypothesis testing (pg15-16).

*R. We agree with the reviewer that we should have included this correction. We have revised our statistical models of this section to use multiple testing correction (Benjamini Hochberg, FDR).*

***Results L353-357: Next, we looked if there was a relation between dosage compensation and a set of biological and physicochemical protein properties to identify more subtle patterns. Specifically, we checked 5 protein properties for their relationship with protein dosage compensation using AN(C)OVA (Suppl. Table S1.E). Transcript abundance was added as a covariate to the model, as dosage compensation was larger at higher differential transcript levels (Adj p = 2.4x10^-4^). The variables ‘membrane protein’ (yes or no) (FDR adjusted p = 0.696), the number of protein interactions (Adj. p = 1), protein length (a proxy for transcript and protein size) (Adj. p = 1) and hydrophobicity (Adj. p = 1) had no significant relationship with dosage compensation. In contrast, the subcellular location (Adj. p = 0.032) of a protein was significantly linked to its degree of dosage compensation. To investigate this further, we investigated the relation between each subcellular compartment and the compensation rate of its proteins (Suppl. Table S1.E). Cytoplasmatic proteins (n = 39) were significantly less compensated compared to all other proteins (Adj. p = 0.011). Thus, cytoplasmatic proteins follow aneuploidy-induced dosage changes more closely than proteins from other cellular compartments. In contrast, proteins associated with the Golgi apparatus (n = 2) had a significantly higher compensation than all other proteins (Adj. p = 0.0065). However, this result needs to be interpreted with caution as the sample size was only 2 proteins. These two proteins were a vesicle-associated membrane protein and a small GTP-binding protein Rab18, both known to be secreted. Proteins from mitochondria (Adj. p = 0.32, n = 18), nucleus (Adj. p = 0.50, n = 13), peroxisome (Adj. p = 0.81, n = 6) and ER (Adj. p = 0.74, n = 2) did not have significantly different compensation rates compared to proteins from all other compartments.”***

**3.5** Line 352, "extend" should be "extent".

*R. We thank the reviewer for finding this typo and have corrected it in the manuscript.*

**3.6** Line 530: "...these aneuploidy-induced changes can drive metabolomic differences between Leishmania strains." Aneuploidy-induced changes to transcripts and proteins were shown to correlate with a subset of metabolites. You did not prove that these changes in transcript and protein expression drove metabolic differences. Please revise this concluding statement.

*R. The reviewer is correct that we did not show causality, but only a correlation. We have adapted the sentence accordingly.*

***Conclusion L588-L590: “*In *Leishmania* strains with a closely related genetic background, the degree of these aneuploidy-induced changes ultimately correlates with the degree of metabolomic difference”**

**3.7** I don't understand the meaning of the statement "widespread transcript regulation modulating aneuploidy-related stress was absent" - please revise or clarify (pg 2).

*R. We have now clarified this sentence in the abstract.*

***Abstract L33-L35: “In contrast to other Eukaryotes, we did not observe the widespread regulation at the transcript level that typically modulates some of the negative effects of aneuploidy”. This is more similar to how we also explain it in the Discussion section.***

**3.8** Much of the work in this paper is consistent with findings that have been previously conducted in other organisms, and these papers should be cited accordingly. The effects of aneuploidy in trans were first described by the Amon Lab in PMID: 22802626. The role of complex subunit assembly in aneuploidy-associated dosage compensation was described in PMID: 27720452. A recent preprint has also examined the effects of aneuploidy on protein expression in cancer - <https://www.biorxiv.org/content/10.1101/2021.06.18.449005v1> - how similar are your results to the analysis presented there?

*R. We thank the reviewer for this comment, which was also made by reviewer 2. Dosage compensation of protein-complex subunits and trans-effects have indeed been shown before in other Eukaryotes. We already mentioned this in the manuscript and provided several references for this, but have now supported it with additional context, comparison, and references (including also* PMID: 22802626 and PMID: 27720452) *as per the comments of reviewer 2 (see comments 2.3, 2.4, 2.5, 2.6).*

**3. Final** Reviewer #3 (Significance (Required)):

My expertise is in cancer genomic instability, and I am not an expert in Leishmania. Therefore, I feel only moderately qualified to assess the significance of this manuscript.

On a basic level, the results reported herein largely recapitulate prior observations made in aneuploid yeast, mouse, and human cells. EG, extensive dosage compensation of protein complex subunits encoded on aneuploid chromosomes was first shown in yeast (PMID: 20850176 and 25073701) and then later extended to cancers as well (PMID: 22968442 and [https://doi.org/10.1101/2021.06.18.449005](https://eur01.safelinks.protection.outlook.com/?url=https%3A%2F%2Fdoi.org%2F10.1101%2F2021.06.18.449005&data=04%7C01%7CBart.cuypers%40uantwerpen.be%7C6b1eceb5d5de453bf16208d9ad934900%7C792e08fb2d544a8eaf72202548136ef6%7C0%7C0%7C637731672653085481%7CUnknown%7CTWFpbGZsb3d8eyJWIjoiMC4wLjAwMDAiLCJQIjoiV2luMzIiLCJBTiI6Ik1haWwiLCJXVCI6Mn0%3D%7C3000&sdata=3xCWD2LI1NwFPx6YP7mShOmQYlVU%2BV2Nd%2B3CKY2IUCo%3D&reserved=0)). While the authors emphasize the odd lack of transcriptional regulation in Leishmania, it has been appreciated that aneuploidy results in major post-transcriptional regulation in other organisms as well. So, from that perspective, I see this paper as an extension of what's been observed in other systems.
However, I am not an expert in Leishmania, and I hope that other reviewers will comment on the extent to which this paper would represent a significant advance in the study of this organism in particular.

*R.* *We thank the reviewer for this honesty and transparency. We indeed believe our paper provides a significant advance towards understanding the molecular impact of aneuploidy in Leishmania, but also for Trypanosomatids and the Euglozoid phylum in general. To our knowledge, this is the very first time the impact of aneuploidy in these organisms has been studied on the proteome and metabolome levels. Additionally, we link these data to a comprehensive transcriptome analysis on exactly the same samples. The insights provided by our manuscript are essential to understand the molecular consequences and function of mosaic aneuploidy in Leishmania where hundreds to thousands of karyotypes can co-exist (and potentially drive rapid adaptation), as has recently been demonstrated by a single-cell genomics study from our group (Neigreira et al. 2022 NAR: PMID: 34893872).*
*It is true that we see several parallels with other Eukaryotes despite the completely different gene regulation in Leishmania, and elaborately point this out in the manuscript. We are convinced there is significant value in reporting on the molecular, systems biological impact of aneuploidy in this branch of understudied Eukaryotes, whether these are similarities or differences. However, we do also highlight differences with other Eukaryotes (see also comment 1.5 where reviewer 1 remarks on the novelty of trans-proteomic changes without trans-transcriptome changes and “1. Final”)* *as well as novel observations (compensation of cytoplasmic proteins, strong relation between degree of transcriptome/proteome changes and metabolome changes).*
