# Supplementary material for: Four layer multi-omics reveals molecular responses to aneuploidy in Leishmania
Source: PLoS Pathog. 2022 Sep 23;18(9):e1010848. doi: 10.1371/journal.ppat.1010848 (PMC9534393; doi:10.1371/journal.ppat.1010848)
Supplement: Attachment — Submitted filename: Rebuttal_Letter.docx [file ppat.1010848.s008.docx]

# Four layer multi-omics reveals molecular responses to aneuploidy in *Leishmania*

**Rebuttal Letter**

**Part I – Summary**
Please use this section to discuss strengths/weaknesses of study, novelty/significance, general execution and scholarship.

**Reviewer #1:** The revisions have adequately addressed my concerns, and I support publication of this manuscript in its current form.

RI.1 We thank the reviewer for the constructive feedback in the previous round of peer-review.

**Reviewer #2:** This manuscript reveals that Leischmania can adapt to new environments via gene dosage effects. This can occur because the species has a large number of chromosomes that divide the genome into many segments and because of its constitutive mode of gene expression. This is an interesting case and worthy of publication following some revisions.

RI.2 We thank the reviewer and have made the requested revisions **(See Part II.2)**

**Reviewer #3**: The authors addressed all my comments and explained well their reasoning in most cases, particularly the method details, with one notable exception. In the paragraph clarifying the novelty for eukaryotes, I am still not sure whether I understand their interpretation of their data and the comparison to other eukaryotes.

RI.3.1 We thank the reviewer for the constructive feedback in the previous round of peer-review. We agree with this final concern and have modified the paragraph in question accordingly (see directly below).

Below is the text what the authors now included in discussion. They state that most aneuploids display regulation of trans-transcripts, while Lieshmania execute most effects via regulation of proteins in trans. The authors first need to clearly define the terms trans-trancript and trans-protein.

RI.3.2. We defined the terms extensively in Results L378-L386, but agree their usage in the Conclusion section could lead to confusion. We have omitted the terms trans-transcripts and trans-proteins from the Conclusion section.

**Removed:** Original manuscript L596-L598 “Instead, Leishmania features a surprisingly high number of trans-proteins, which are likely post-transcriptionally modulated. It thus seems that aneuploidy is accompanied by trans-proteomic changes in Eukaryotes, regardless of the system of regulation. Post-transcriptional modulation of trans-proteins”

Further, the difference is not all that clear. The current model is rather that the proteotoxic stress impairs many aspects of cellular physiology, and in reaction to that specific stress response pathways – autophagy, unfolded protein response, inflammatory response etc – are activated. Not all of these responses occur via activation of transcription. Some proteins get stabilized or activated (eg. proteasome proteins, autophagy), others degraded (replication factors). Thus, I think the authors should attempt a more differentiated account of the response to aneuploidy. Finally, as already stated before, why is it such a surprise that Leishmania differentially regulates the proteins and not the transcripts in response to aneuploidy? The authors explain already in the beginning of the manuscript that this is the way how Leishmania regulates gene expression. Leishmania just simply cannot do it differently. By the way, I also believe that Leishmania is a very attractive model, but some of the arguments used here are not entirely clear and correct.

RI.3.3. We agree with the reviewer that the difference with other Eukaryotes was not clearly described in the previous version of the manuscript. In addition, reviewer 2 remarked that we do not have sufficient evidence to demonstrate a fundamental difference with other Eukaryotes. Therefore, we have removed these claims from the manuscript.

Indeed, we have now largely rewritten the conclusion:

*Original Manuscript L593-L602: “While most aneuploid Eukaryotes display extensive regulation of trans-transcripts to mediate the effects of aneuploidy, we did not observe this to the same extend in Leishmania. This suggests that the post-transcriptional mRNA regulation system of the parasite does not mediate the impact of aneuploidy. Instead, Leishmania features a surprisingly high number of trans-proteins, which are likely post-transcriptionally modulated. It thus seems that aneuploidy is accompanied by trans-proteomic changes in Eukaryotes, regardless of the system of regulation. Post-transcriptional modulation of trans-proteins may also be present in other Eukaryotes, but could be overshadowed by the transcription factor-induced gene regulation that is absent in Leishmania. For this reason, we believe that Leishmania is an attractive model to study processes of posttranscriptional modulation of protein levels in the context of aneuploidy and beyond.”*

*To:*

*Modified Manuscript L609-614: “Differentially expressed transcripts and proteins between aneuploid Leishmania strains also originated from non-aneuploid chromosomes. At protein level, these were enriched for proteins involved in protein-metabolism, including chaperones and chaperonins, peptidases, and heat-shock proteins. We believe that the high karyotype diversity in vitro and absence of classical transcriptional regulation make Leishmania an attractive model to study processes of protein homeostasis in the context of aneuploidy and beyond.”*

With regard to the reviewer’s comment: “*Finally, as already stated before, why is it such a surprise that Leishmania differentially regulates the proteins and not the transcripts in response to aneuploidy? The authors explain already in the beginning of the manuscript that this is the way how Leishmania regulates gene expression. Leishmania just simply cannot do it differently.”*

Despite the polycistronic and constitutive transcription, final mRNA levels can be post-transcriptionally regulated in *Leishmania* (e.g. by alba-domain proteins). Therefore, we needed to consider this at least as a theoretical possibility in the manuscript. We now clarify this better and in the discussion of the manuscript:

L583-L587: *Here, in Leishmania, we found relatively low proportions of differential trans-transcripts and no significant enrichment of specific functional classes amongst these. While Leishmania lacks transcriptional regulation of individual protein-coding genes, a more extensive regulation would have been a theoretical possibility, as post-transcriptional regulation of individual transcripts (eg. by alba-domain proteins) was observed in the context of parasite development^66^.*

In addition, we have removed speculation about (the lack of) trans-transcript regulation to mitigate the impact of aneuploidy from the conclusion (also original manuscript L593-L602)*.*
Below is the paragraph in question, with some remarks and underscored unclear
Novelty for other Eukaryotes:
Conclusion L593-L602: “While most aneuploid Eukaryotes display extensive regulation of trans-transcripts to mediate the effects of aneuploidy, we did not observe this to the same extend in Leishmania (the authors do not show any comparisons of the “extends”). This suggests that the post-transcriptional mRNA regulation system of the parasite does not mediate the impact of aneuploidy (to my knowledge post-transcriptional regulation of mRNA – do they mean mRNA stability? - has not be observed in any other aneuploids). Instead, Leishmania features a surprisingly high number of trans-proteins, which are likely post-transcriptionally modulated. It thus seems that aneuploidy is accompanied by trans-proteomic changes in Eukaryotes, regardless of the system of regulation. Post-transcriptional modulation of trans-proteins (I am sorry but I am really not sure what this means) may also be present in other Eukaryotes, but could be overshadowed by the transcription factor-induced gene regulation that is absent in Leishmania (Do the authors mean that it is impossible to recognize whether protein abundance was regulated on transcript or protein level? That’s incorrect. This data is available for other organisms and have been analyzed). For this reason, we believe that Leishmania is an attractive model to study processes of posttranscriptional modulation of protein levels in the context of aneuploidy and beyond.”

RI.3.4. See RI.3.3 where this section was completely revised. Additionally, “to the same extend” was removed and replaced by “coordinated transcriptomic response”. Post-transcriptional regulation was removed.

**Part II – Major Issues: Key Experiments Required for Acceptance**
Please use this section to detail the key new experiments or modifications of existing experiments that should be absolutely required to validate study conclusions.

Generally, there should be no more than 3 such required experiments or major modifications for a "Major Revision" recommendation. If more than 3 experiments are necessary to validate the study conclusions, then you are encouraged to recommend "Reject".

Reviewer #1: No major issues

Reviewer #2: While this is a revised version from a previous review, there are some aspects that need further revision. In some cases, the authors did not seem to assimilate the suggestions about underlying principles in general and just Band Aided over some issues. In some aspects the authors might be trying too hard to claim novelty, which is not really necessary—the findings are interesting without embellishment. The Abstract and the text states that dosage compensation was found for protein complex subunits and non-cytoplasmic proteins. However, only 5 out of 32 analyzed show no change compared to the normal. Thus, this is a stretch. The basis of this is probably trivial: excess subunits for complexes are just degraded. There is no evidence provided for an active process of dosage compensation. The authors need to remove the term dosage compensation from the manuscript and instead refer to the situation as protein subunit homeostasis or something else but not dosage compensation. A claim of some dosage compensation process is not needed for the significance of the study.

R.II.2.1 We thank the reviewer for this comment. We did not imply any form of active compensation, but indeed rather passive forms like protein subunit homeostasis. We intended to indicate that some types of proteins had significantly lower abundance levels that would have been expected by their aneuploidy-induced gene dosage changes. We acknowledge that the term ‘dosage compensation’ is primarily used for active processes in literature, and might therefore be confusing to the readers. We therefore removed ‘dosage compensation’ and ‘compensation’ throughout the manuscript. We now refer instead to proteins with ‘a reduced dosage response’ or ‘attenuated proteins’, indicating that the protein subsets have lower abundances than expected by dosage, but without claiming anything about the underlying process (active or passive) for this.

The second stretch is the authors state that the effects of aneuploidy are fundamentally different than in other eukaryotes. However, the aneuploids analyzed are for small chromosomes because larger chromosomes are not found in an aneuploid state. This is the case even though the larger chromosomes are still smaller fractions of the whole genome than individual chromosomes in most other species. This suggests that larger aneuploids are indeed detrimental. The absence of their study leaves a void in terms of why that is the case. These claims should be removed from the manuscript because the study was not comprehensive enough to support this claim.

R.II.2.2 We agree with the reviewer that a more careful interpretation of our data with regards to fundamental differences with other Eukaryotes is due.

This difference with other Eukaryotes was discussed in the Discussion and the Conclusion.

For the Conclusion: We have noted the same concern from reviewer 3, and have now completely rewritten the conclusion. See RI.3.3. In the new version, we do not claim any fundamental difference anymore with other Eukaryotes in how the impact of aneuploidy is regulated.

For the discussion, we did the same by removing the following section:
**Removed:** Discussion: Original Manuscript L574-578 “This direct modulation at protein-level (without underlying transcriptional change) may in fact also be present in other Eukaryotes, but could be overshadowed by the transcription factor-induced changes. For this reason, we propose *Leishmania* as an attractive model to study processes of post-transcriptional modulation of protein levels in the context of aneuploidy.”

The issue is raised that mRNAs and protein levels do not necessarily correspond. This is widely known for steady state levels of most gene products with the exception of transcription factors and some other proteins (Ori et al., 2016, Genome Biol 17:47). Thus, the data presented do not provide evidence of any active process that accounts for the differences. The discussion of this topic should not be emphasized as particularly novel.

R.II.2.3. We agree with the reviewer and have removed the following sentence from the discussion to put less emphasis on this observation.

**Removed:** Discussion: Original Manuscript L573-L574: “… and suggests a similar mediation of aneuploidy-induced effects as in other Eukaryotes, but instead regulated directly at the protein level.”

Reference 1 (Siegel and Amon) should be replaced with Birchler and Veitia, 2021, Cytogenetics and Genome Research 161: 529-550, which is more recent, more historically accurate, more comprehensive, and illustrates how the effects of aneuploidy intersect with other biological and physical chemistry principles.

R.II.2.4. We have replaced the reference accordingly.

Specific comments:

Lines 377 to 378: As noted previously, these references do not support the statements in this sentence. In the response, the authors say they substituted these for Sun et al, Hou et al., Raznahan et al, and Zhang et al, but there is no change. The references 46-49 should be deleted. Indeed, Hou et al showed that 47 and 49 needed re-interpretation. Reference 46 (Hwang et al) claims there are few trans effects but examination of their data shows they are abundant.

R.II.2.5. We apologize for this error. We have now replaced the reference accordingly (L398-400 of the new manuscript).

As noted in the previous review, single celled organisms such as yeast can select changes in absolute dosage to increase flux through certain pathways (Conant and Wolfe, 2007; Mol Syst Biol 3: 129). The case here of Leschmania may have some parallels to this situation, where there is polymorphism for aneuploid chromosomes that might provide a selective advantage in some circumstances. Nevertheless, under most circumstances, aneuploidy in yeast is detrimental and there is evidence for dosage sensitivity of the regulatory machinery that produces trans effects across the genome. Claims that there are few trans effects in yeast (mainly from the Amon group) are not supported by the fact that dosage sensitive genes were retained following the whole genome duplication in the evolutionary past of this species and are underrepresented in CNV of yeast (Hakes et al 2007, Genome Biology 8: R209) and studies of haploinsufficiency (Papp et al Nature, 2003) and overexpression (Robinson et al 2021). The dosage sensitive genes are typically members of multicomponent regulatory complexes (and some others). Thus, there are indeed dosage effects of aneuploids and selected genes in yeast. Now, it is potentially possible that the constitutive expression in Leischmania has evolved a different process. However, the fact that larger aneuploids do not appear to be so polymorphic as for the shorter chromosomes and were not studied suggests that the available data cannot support a claim that there is a difference. Further study would be needed but is beyond the scope of this submission.

R.II.2.6. We thank the reviewer for these insights which provide interesting avenues for our future research.

In the previous revision round, we followed the argumentation of the reviewer that the fact that smaller chromosomes are more frequently aneuploidy in *Leishmania* (just as in yeast and like chromosome 21 in human), might be a sign that aneuploidy is to some extend detrimental to the parasite. Reading the argumentation above, we agree with the reviewer that the situation might indeed be similar as in yeast where fitness benefits of aneuploidy must outweigh the fitness costs in a given environment.

We have now added a final sentence to the following paragraph to reflect this better:

L539-L547: “It is possible that this stochiometric imbalance caused by aneuploidy is too some extent detrimental to the parasite. One argument in this direction is that we and previous studies found smaller *Leishmania* chromosomes (with smaller numbers of genes) to be more frequently aneuploid, which matches observation in yeast and humans (chromosome 21 has the smallest number of genes in humans and the least severe autosomal aneuploid)^11,24,61^. Additionally, it has been shown in yeast that genes that are deleterious when overexpressed are enriched for protein complex subunits^62^. **From this perspective, the situation in *Leishmania* could be similar to other single-celled organisms such as yeast, where the fitness benefits of aneuploidy must outweigh the fitness costs in a given environment** ^62^.”

Reference 62 is Robinson et al 2021, which the reviewer suggested.

The authors need to pull back from claims of some type of active dosage compensation and of a fundamental difference with other eukaryotes because the data do not support these claims or at least leave ambiguity. The documentation that there is polymorphism for several shorter chromosome aneuploidies that could potentially have adaptive roles is sufficiently important and interesting on its own.

R.II.2.7. We agree with the reviewer that there is not sufficient evidence for active dosage compensation or fundamental differences with other Eukaryotes. We have modified the manuscript accordingly (see See R.II.2.1 for active dosage compensation and See RI.3.3, R.II.2.2, See R.II.2.3 for differences with other Eukaryotes).

Reviewer #3: (No Response)

**Part III – Minor Issues: Editorial and Data Presentation Modifications**
Please use this section for editorial suggestions as well as relatively minor modifications of existing data that would enhance clarity.

Reviewer #1: No major issues

Reviewer #2: See above.

Reviewer #3: (No Response)
